# Cytogenetic and molecular analysis of distal 4q duplication with distinctive phenotype using single-nucleotide polymorphism array

**DOI:** 10.1186/s13039-021-00568-9

**Published:** 2021-09-29

**Authors:** Jianlong Zhuang, Na Zhang, Wanyu Fu, Jianfeng Yao, Yanqing Li, Shuhong Zeng, Yuanbai Wang, Yingjun Xie, Yuying Jiang

**Affiliations:** 1 Prenatal Diagnosis Center, Quanzhou Women’s and Children’s Hospital, Quanzhou, 362000 Fujian People’s Republic of China; 2 Department of Women Healthcare, Quanzhou Women’s and Children’s Hospital, Quanzhou, 362000 Fujian People’s Republic of China; 3 Department of Obstetrics and Gynecology, Key Laboratory for Major Obstetric Diseases of Guangdong Province, Key Laboratory of Reproduction and Genetics of Guangdong Higher Education Institutes, The Third Affiliated Hospital of Guangzhou Medical University, Guangzhou, 510150 Guangdong People’s Republic of China

**Keywords:** Partial trisomy 4q, 6p deletion, Xp deletion, Karyotype analysis, SNP array

## Abstract

**Aims:**

There is little knowledge about partial trisomy 4q and the genotype–phenotype correlation. In this study, we presented the detail of two Chinese families with partial distal 4q duplication in an attempt to clarify the correlation between the genotype and the phenotype.

**Methods:**

Two pedigrees with distal 4q duplication were enrolled in this study. Karyotype analysis and single-nucleotide polymorphism (SNP) array detection were performed for prenatal diagnosis. Fluorescence in situ hybridization analysis. (FISH) was conducted to verify the copy number variants.

**Results:**

Two families with partial trisomy 4q were identified. The fetus in pedigree 1 exhibited multiple ultrasound anomalies including intrauterine growth restriction and an atrioventricular septal defect who had a duplication of 4q28.3-qter associate with 6p25.2-p25.3 deletion, which resulted from balanced translocation carried by his father t(4;6)(q28.3;p25.2). The fetus in pedigree 2 had a distal 4q28.3-qter duplication combined with monosomy of Xp21.3-p22.3, and the karyotype was described as 46,X,der(X)t(X;4)(p21.3;q28.3)mat, which originally inherited from the pregnant woman who exhibited a mild clinical phenotype limited to short stature.

**Conclusions:**

In our study, we for the first time identified the partial trisomy 4q associate with 6p or Xp deletion. In addition, our finding further strengthens that mild clinical phenotype in 4q duplication case may be due to the spreading of X inactivation to the autosomal in derivation of chromosome X.

## Introduction

Over 60 cases of partial trisomy 4q have been reported since it was first identified by Hoehn et al. in 1971 [1]. According to current knowledge, most patients with partial trisomy 4q derive the unbalanced chromosome abnormities from one of their parents who carries balanced translocation, which is always accompanied with partial monosomy of another chromosome. It is therefore difficult to delineate the genotype–phenotype correlation because of the combination in partial monosomy of the chromosome [2, 3]. Partial trisomy 4q is less common and has variable manifestations including developmental delay, intellectual disability, microcephaly, low-set ears, broad nasal bridge, and congenital heart defects [4].

Deletion involving chromosome 6p25 has been defined as a clinically identifiable syndrome, which was first described by Ritscher et al. in 1987 [5]. So far, over 68 cases of monosomy 6p25 syndrome have been reported [6], and most of them manifested variable clinical features including developmental delay/intellectual disability, language impairment, hearing loss, and ophthalmologic, cardiac, and craniofacial abnormalities [7]. Among them, ocular and hearing abnormalities are the most remarkable features [8]. A previous study suggests that the mutation of *FOX* group may be the most critical factor to the clinical phenotype [9].

Chromosome Xp deletions are common aberrations exhibiting variable features. Distal deletions of the Xp usually present with limited phenotypes in females, which may be due to X-chromosome inactivation [10]. Defects in the* SHOX* gene in this region are also seen in LeriWeill dyschondrosteosis, a skeletal dysplasia characterized by a short stature, meromelia, and Madelung wrist deformity [11]. In addition, previous studies have documented that deletions or mutations of the* STS* gene on Xp region are associated with X-linked ichthyosis, a dermatologic disorder presenting with dry, scaly skin due to steroid sulfatase deficiency [12].

In the present study, we identified two rare cases of partial trisomy of 4q associated with other monosomy chromosomes. One case involved a patient who carried a duplication of 4q28.3-qter and microdeletion of 6p25.2-q25.3, which has never been described before. The other involved a fetus and its pregnant mother, both of whom carried partial trisomy 4q with Xp deletion, representing the mild clinical phenotype. In addition, the clinical features described in previously reported cases involving distal 4q duplication were also reviewed (Table 1).

**Table 1:** Comparison of the clinical findings in patients with parital trisomy 4q

	Taylor et al. [13]	Stoll et al. [14]	Mikelsaar et al. [15]	Lundin et al. [16]	Rinaldi et al. [17]	Lin et al. [18]	Egritas et al. [19]	Velinov et al. [20]	Cakmak−Genc et al. [21]	White et al. [22]	Our study	
											Case 1	Case 2
Duplication region	4q26-q35	4q26-qter	4q25-qter	4q27-q35	4q24-q35	4q28.1-q35	4q25-qter	4q25-qter	4q28-qter	4q24-qter	4q28.3-qter	4q28.3-qter
Deletion region	−	−	−	−	−	−	−	21q21.1-q22.3	9p24-pter	Xq22-qter	Xp21.3-p22.33	6p25.2-p25.3
Age/Sex	6.5/M	11/F	7/F	13/F	1/M	2.5/M	1.25/F	NB/F	NB/F	32/F	28/F	Fetus
Growth retardation	+	+	+	+	+	+	−	+	NM	−	−	IUGR
Intellectual disability	+	+	+	+	+	−	+	NM	+	−	−	NA
Microcephaly	+	+	+	+	+	−	+	+	−	−	−	NA
Low−set/malformed ears	+	+	+	−	+	+	+	+	+	−	−	NA
Retrognathia/micrognathia	NM	NM	−	−	+	−	+		+	−	−	NA
High/broad nasal bridge	+	+	+	+	+	+	NM	+	+	−	−	NA
Thumb anomalies	−	NM	−	+	+	−	+	NM	−	−	−	NA
Epicanthic folds	NM	NM	NM	+	NM	+	−	+	NM	−	−	NA
Congenital heart defect	+	NM	−	−	+	NM	−	+	+	−	−	+
Renal hypoplasia	NM	NM	−	−	−	+	−	NM	−	−	−	NA
Epilepsy	+	NM	−	NM	−	−	NM	NM	+	−	−	NA
Other		Factor X defect				Choanal-atresia	Cholestasis		Choanal-atresia	Secondary amenorrhea	Short stature	

−:absent; +: present, *NA* not applicable, *NB* new born, *NM* not mention, *F* female, *M* male, *IUGR* intrauterine growth restriction

## Methods

### Subjects

Enrolled in this study were two Chinese pedigrees carrying partial trisomy 4q, and both of them denied any related family history. The two pregnant women involved in this study came to Quanzhou Women’s and Children’s Hospital for prenatal diagnosis because of different high-risk factors. The study was commented upon the approval from the Institutional Ethics Committee of the hospital (2020No.31).

Pedigree 1 involved a 28-year-old pregnant woman (gravida 3, para 1) who gave birth to a female baby 7 years ago with a chromosomal balanced translocation between chromosome 4 and 6. In addition, she aborted a fetus by induction 4 years ago at the 3rd month of pregnancy due to fetal ultrasound abnormalities, including fetal nuchal translucency thickness (9 mm), fetal edema and cervical hydrocystoma. In this pregnancy, fetal ultrasound scan revealed that the fetus had an atrioventricular septal defect, coronary sinus dilation, a left ventricular punctate hyperechoic lesion, a small amount of pericardial effusion, and fetal growth restriction at gestational age of 22^+6^ weeks. Invasive prenatal diagnosis was then recommended and conducted at gestational age of 23^+6^ weeks through karyotype and SNP array analysis. The parents in this family were healthy and denied any consanguinity.

Pedigree 2 involved a 29-year-old pregnant woman (gravida 2, para 0). Stillbirth occurred at 6 months in the first pregnancy in 2019, for which no chromosomal examination was performed. In this pregnancy, non-invasive prenatal testing (NIPT) was performed and the result indicated duplication of chromosome 4 and deletion of chromosome X in the fetus. Meanwhile, prenatal fetal ultrasound scan showed smaller femurs. Physical examination of the pregnant woman and her mother showed a mildly short stature (150 cm in height), and no other significant abnormalities otherwise. The kidneys and heart ultrasound of the pregnant woman and her mother were normal. In addition, an older brother of the pregnant woman died at 1^+^ month after birth with no genetic examination done. Karyotype and SNP array analyses were performed using amniotic fluid cells obtained by amniocentesis. In addition, the parents of the family denied any consanguinity.

### Cytogenetic analysis

Approximately 20 ml amniotic fluid was obtained by amniocentesis for fetal karyotype analysis. Approximately 2 ml parental peripheral blood was collected from the fetus for karyotype analysis. The cultured amniotic fluid cells and peripheral blood lymphocytes were harvested using a SinochromeChromprepII automatic chromosome harvesting system according to the standard protocol (Shanghai Lechen Biotechnology Co., Ltd.). Twenty metaphases were analyzed for peripheral blood karyotype, and 30 metaphases were analyzed for fetal chromosomal karyotype. Nomenclature of chromosomal karyotype refers to ISCN 2020.

### SNP array detection

DNA was extracted from amniotic fluid cells and peripheral blood with QIAamp DNA Blood Kit (QIAGEN, Germany) according to the manufacturer’s protocol. The genomic DNA was subsequently digested, ligated, PCR amplified, purified, fragmented, labeled and hybridized according to Affymetrix CytoScan Assay USER GUIDE using the Affymetrix Cytoscan 750 K array. Genotyping Console and Chromosome Analysis Suite software was used to analyze the SNP and copy number variants (CNVs). The pathogenicity of the CNVs were interpreted according to the standards and guidelines released by the American College of Medical Genetics [23]. Databases of DGV (http://dgv.tcag.ca/dgv), OMIM (https://omim.org/), DECIPHER (https://decipher.sanger.ac.uk/) and PubMed (https: //www.ncbi.nlm.nih.gov/ pubmed/), together with other databases, were used as reference resources.

### Fluorescence in situ hybridization analysis

Amniotic fluid culture cell suspension was used to make slides, which were subsequently denatured. The probes (Aneu Vysion Multicolor DNA probe kit) were selected according to the results of SNP array detection. The slides were hybridized, eluted, counterstained, covered with the cover glass, placed in the dark for 10–20 min, observed for FISH signals and photographed under an inverted fluorescence microscope.

## Results

**Fig. 1:**
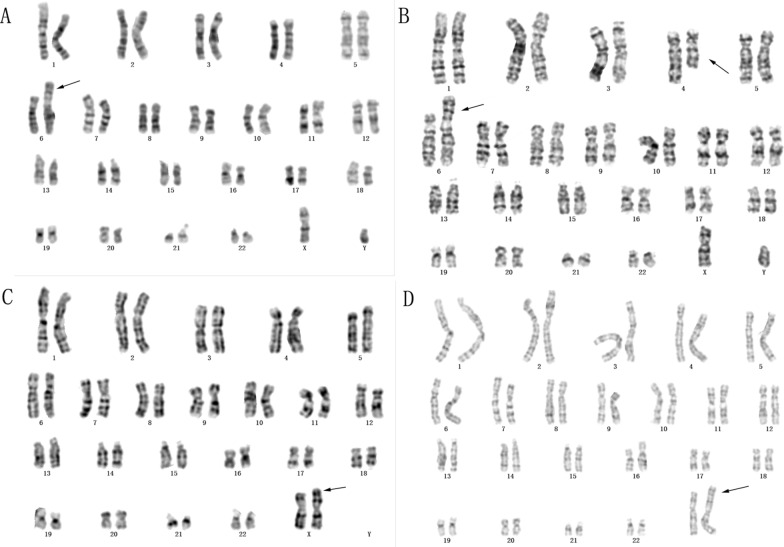
The results of karyotype analysis in the two pedigrees. The arrow indicates chromosomal abnormalities. **A**: A derivation chromosome was shown in chromosome 6 in the fetus of pedigree 1. **B**: A balanced chromosome translocation was observed in the fetus’s father in pedigree 1. **C**: A derivation chromosome X was depicted in the fetus of pedigree 2. **D**: Karyotyping analysis showed that the pregnant woman in pedigree 2 carried the same derivation chromosome

The chromosomal karyotype analysis of the fetus in pedigree 1 showed a derived chromosome 6. Subsequent karyotype analysis in the other family members of pedigree 1 revealed a balanced translocation between chromosome 4 and 6 in the father and his elder sister, which was initially described as t(4;6)(q27;q23). The results indicated that the derived chromosome 6 in the fetus inherited from his father and therefore the fetal karyotype was described as 46,XY,der(6)t(4;6)(q27;q23)dpat (Fig. 1).

**Fig. 2:**
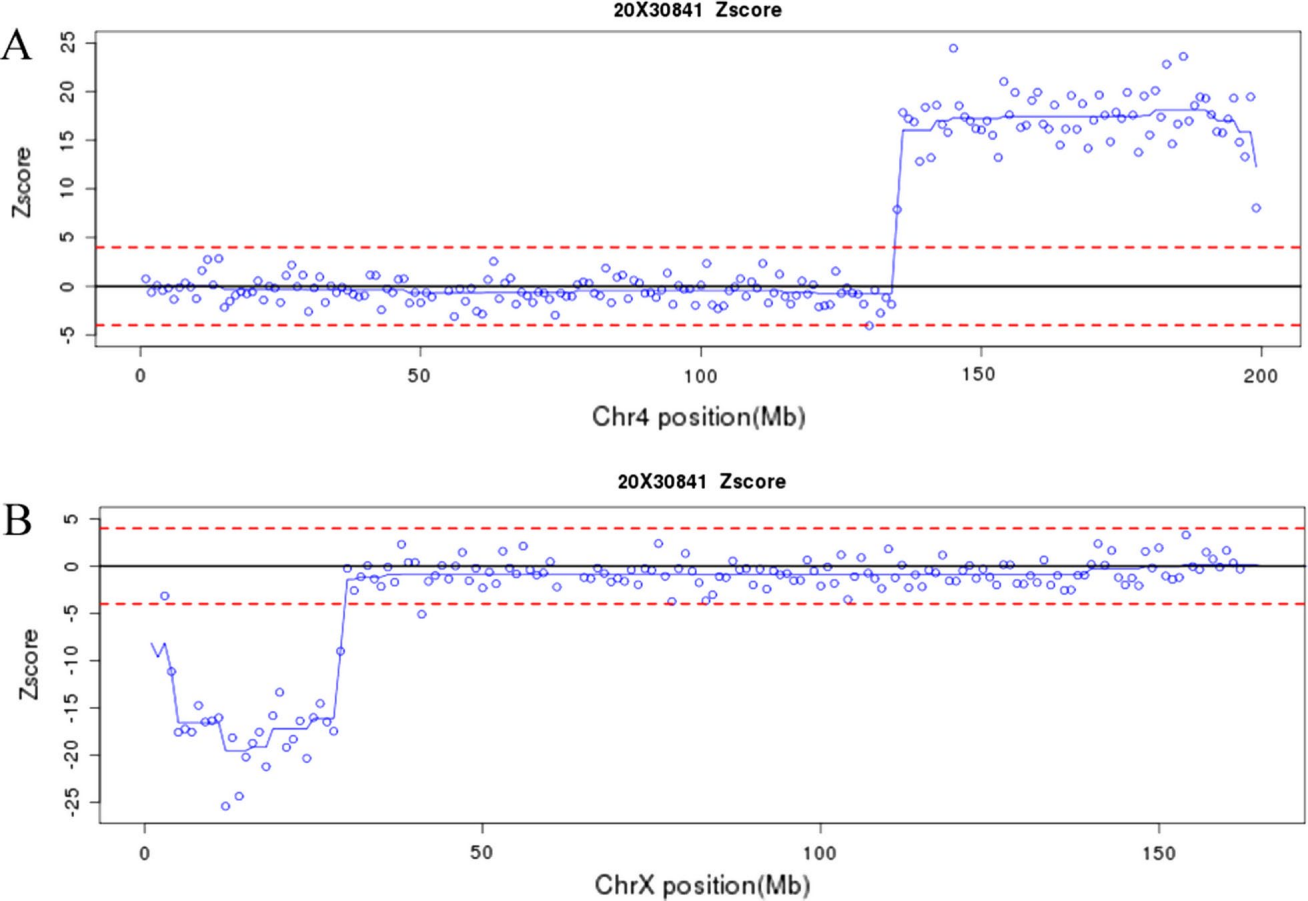
The NIPT results in the fetus of pedigree 2. The results of NIPT screening indicated a duplication of chromosome 4 and a deletion of chromosome X. **A**: The result showed a higher Z-score in distal chromosome 4, indicating a duplication of 4q in the fetus. **B**: A lower Z-score was observed in chromosome Xp, suggesting a deletion of chromosome Xp

Analysis of the chromosomal karyotype in the fetus of pedigree 2 elicited a derived chromosome of chromosome X. Further parental chromosome karyotype analysis showed that the pregnant woman and her mother had the same abnormal chromosome, which was preliminary described as 46,X,der(X)t(X;?)(p22;?) (Fig. 1). Additionally, NIPT results in the fetus indicated the duplication of chromosome 4 and deletion of chromosome X (Fig. 2). Thus, derived chromosome X may compose from partial chromosome 4 and X, although it requires further confirmation.

**Fig. 3:**
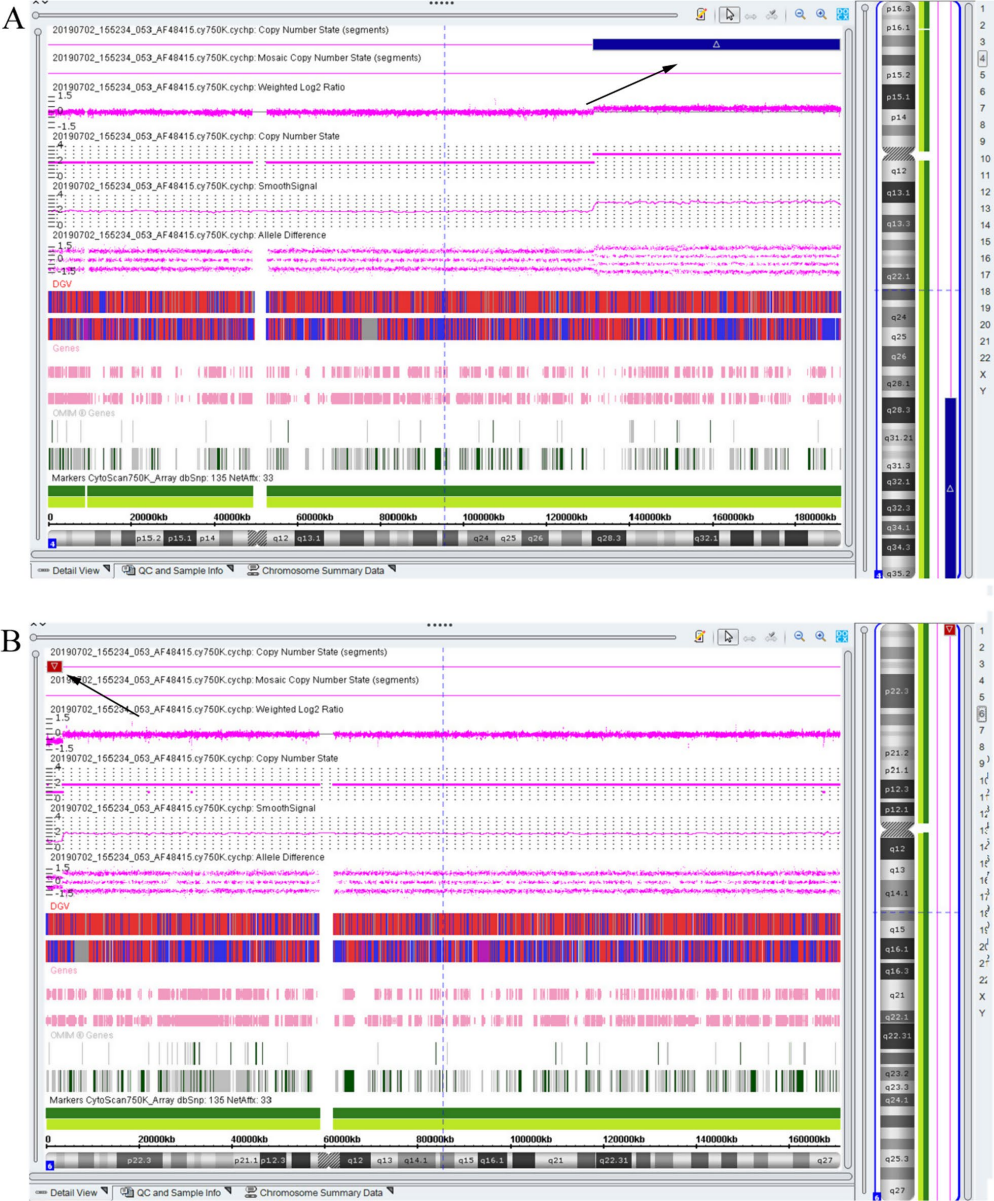
The results of SNP array detection in the fetus of pedigree 1. **A**: A duplication of chromosome 4 was observed; the arrow indicates the duplication region. **B**: A deletion of chromosome X was detected; the arrow indicates the deletion locus

In the study, SNP array detection was performed to analyze the CNVs and confirm the breakpoint of chromosomes. The SNP array detection result of the fetus in the pedigree 1 showed a 59.5-Mb duplication fragment at 4q28.3-qter region (arr[hg19] 4q28.3q35.2(131,389,050–190,957,460)) (Fig. 3), 136 OMIM gene were covered including *TLL1*, *VEGFC* genes. As delineated in the DECIPHER database, the duplication fragments, which was lesser or similar to our case, showed a variety of abnormalities in the phenotype (DECIPHER ID: 285956, 21109, 280404, 256294), including intrauterine growth retardation, global development delay, abnormality of prenatal development or birth, aplasia/hypoplasia of the thumb, and cystic hygroma. Meanwhile, the SNP array results showed that the fetus had a 3.1-Mb deletion in the 6p25.2p25.3 region of chromosome 6 (arr[hg19] 6p25.2p25.3 (376,722–3,552,492) (Fig. 3), which contained 18 OMIM genes including *FOXC1* gene. 6p25 deletion syndrome was identified, with ocular and hearing abnormalities are the most remarkable features. Moreover, none of the detected variants were observed in the DGV database and both of the CNVs were absent in both parents of pedigree 1. Finally, the two CNVs of the fetus in pedigree 1 were interpreted as pathogenicity. The karyotype of the fetus was finally confirmed to be 46,XY,der(6)t(4;6)(q28.3;q25.3)dpat.

**Fig. 4:**
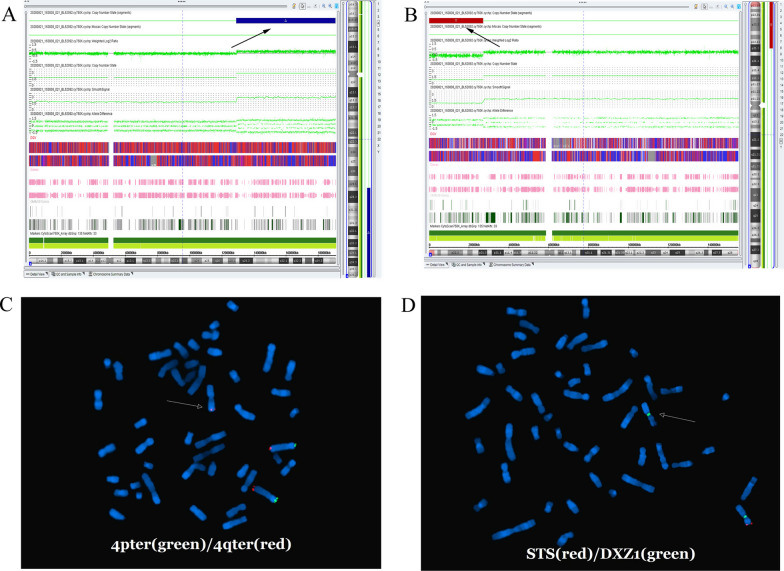
CNVs detection by SNP array and further confirmation by FISH in pedigree 2. **A**, **B**: The results of SNP array detection showed a duplication of chromosome 4 and a deletion of chromosome X in the fetus; the arrow indicates the duplication/deletion region. **C**, **D**: The CNVs verified by FISH analysis. A gain of chromosome 4qter and a loss of STS gene in chromosome Xp were observed in c and d, respectively. The arrow indicates the derivation of chromosome X in the fetus

The SNP array detection results in pedigree 2 elicited that the fetus had a 61.8-Mb duplication in 4q28.3-qter region of chromosome 4 (arr[hg19] 4q28.3q35.2 (129,108,987–190,957,460)) (Fig. 4), which contained 139 OMIN genes similar to those in the fetus of pedigree 1. In addition, the SNP array results showed that the female fetus had a 27.0-Mb deletion in the Xp21.3-p22.33 region (arr[hg19] Xp21.3p22.33(168,551–27,213,664)) (Fig. 4), covering 117 OMIM genes including *SHOX* and *STS* genes. In the DECIPHER database, smaller deletions than our case in chromosome X showed a variety of abnormalities in the phenotype, including a short stature, intellectual disability, obesity, intrauterine growth retardation (DECIPHER ID: 280,404, 256,291, 285,002, 290,204, 288,686).The SNP array results were confirmed by FISH analysis (Fig. 4). The karyotype of the fetus was described as 46,X,der(X)t(X;4)(p21.3;q28.3)mat according to SNP array results. The parental SNP array verification test showed that the pregnant woman and her mother carried the same chromosomal CNVs as the fetus.

The CNVs in the fetuses of both pedigrees were interpreted as pathogenicity. In pedigree 1, the results of fetal ultrasound examination showed that the fetus had cardiac malformations and a variety of ultrasound soft index abnormalities, which are consistent with the duplication of 4q. Finally, the families chose to terminate of pregnancy. Nevertheless, the female fetus in pedigree 2 may have a mild clinical phenotype, which would be similar to the clinical feature of pregnant woman and her grandmother with the clinical phenotype limited to short stature. The family chose to continue the pregnancy, and a full-term baby girl was born with a normal appearance.

## Discussion

Partial trisomy 4q is a rare chromosomal abnormality which usually manifests as growth retardation, intellectual disability, microcephaly, and facial dysmorphism, and cardiac/renal malformations. In the present study, we identified two families that carried partial trisomy 4q chromosomal abnormalities with various phenotypes. We also reviewed previous findings of partial trisomy 4q that similar to our cases, and and found a relationship between the clinical phenotype and duplication of chromosome at distal 4q.

At present, it remains difficult to clarify the genotype–phenotype correlation in partial trisomy 4q. An overview of the cases of 4q28.3-qter duplications revels that the most common manifestations are growth retardation, intellectual disability, microcephaly, low-set or malformed ears, and high or broad nasal bridge (Table 1). A study has reported that severe clinical effects of 4q duplication, including growth retardation, intellectual disability, microcephaly, facial asymmetry, thumb anomalies, hearing impairment, epilepsy, and congenital heart defect, may relevant to the 4q27-q31 region [24]. The study conducted by Elghezal et al. [3] indicates that the region of 4q31-q33 is critical for the development of characteristic dysmorphic features and the region of 4q35 may be involved in the development of microcephaly and severity of intellectual disability and developmental delay [3]. But another study [25] believed that duplication of the 4q33-4q34 region may be the critical for the remarkable clinical phenotype of 4q duplication syndrome. In addition, the relationship between the renal hypoplasia and genotype remains unclear. Some researchers believe that duplication of the 4q22-q23 correlates with renal hypoplsia [26], while a study conducted by Otsuka et al. [27] argued that renal hypoplasia might be female-prone and probably associate with the duplication of 4q33-q34 region. A recent study [28] with duplication of 4q34.1q35.2 reported their ultrasound findings of kidney abnormality, while a deletion of 7q34q36.3 was also observed [29]. In our research, the fetus in pedigree 1 had no renal substantial abnormality. Review analysis of the clinical findings of some cases with 4q28.3-qter showed that only one case exhibited renal hypoplasia [18]. Another study further supported the previous finding that acrorenal syndrome was related to a more proximal 4q trisomy [3]. Thus, we believe that renal hypoplasia may be relate to proximal 4q duplication. It was found in our study that partial trisomy 4q carried in the fetuas in pedigree 1 was associated with partial monosomy 6p. Some phenotypes cannot be clearly defined in the fetal period, such as intellectual disability and facial features. Prenatal ultrasound indicated that the fetus had intrauterine growth retardation, atrioventricular septal defect, which are consistent with the common clinical phenotype of partial trisomy 4q. Previous studies suggest that the *TLL1* gene in 4q32.3 region plays a variety of roles in mammalian heart formation and it is also essential for ventricular septum formation [30], which is believed to associate with autosomal dominant atrial septal defect type 6 disease. In addition, a study described a patient with 46,XY,dup(4)(q28q35.2) who exhibited developmental delay combined with a heart defect, which is similar to the partial trisomy 4q in our study [2]. Several studies also reported heart anomalies associated with 4q trisomy [13, 20, 21]. In contrast, some other studies maintained that ocular and hearing abnormalities are the most remarkable phenotype of 6p25 deletion, which may correlate with *FOXC1*, *FOXF2* and *SERPINB6* genes [31]. In our opinion, the abnormal phenotype observed in the fetal may be ascribed to partial trisomy 4q.

In the rec(X) phenotype of females, a short stature is usually seen in del(Xp), while ovarian failure is commonly observed in del(Xq) [32]. A previous study [22] identified a case harbored partial trisomy 4q associated with monosomy Xq for the first time, indicating that clinical phenotype limited to secondary amenorrhea indicating the inactivation of der(X). In our study of pedigree 2, the pregnant woman and her mother carried a partial trisomy 4q and associated with Xp21.3p22.3 deletion exhibited a short stature, which may relate to inactivation of der(X) and the deficiency of *SHOX* gene. In addition, the deletion of Xp21.3p22.3 also contains *STS* gene, which is associated with X-linked ichthyosis. Studies have showed that female carriers rarely exhibit a skin phenotype [33, 34], while affected males often present with generalized peeling or exaggerated neonatal desquamation at birth, or shortly thereafter, although some may exhibit a collodion membrane [35]. In this study, the pregnant woman and her mother carried *STS* gene deletion and showed none of the corresponding clinical phenotype, which is consistent with the previous study. The patient in pedigree 2 of our study is the second case of partial trisomy 4q associated with chromosome X, who only exhibited phenotype limited to a short stature, which further supports that 4q duplication on the derived X chromosome would also be inactivated, leading to a mild clinical phenotype due to chromosome X deficiency.

In the current practice, NIPT is an effective prenatal screening tool for fetal aneuploidy detection, and the low coverage maternal plasma fetal free DNA sequencing has a potential ability for investigating large fetal chromosomal deletions and duplications (> 10 Mb) [36]. In the pedigree 2 of our study, NIPT results indicated a duplication of chromosome 4 and a deletion of X chromosome in the fetus, which was verified by SNP array analysis, which further supports the feasibility of expanded NIPT for fetal CNVs screening.

## Conclusion

Limit studies are available on partial trisomy 4q. In this study, we identified two pedigrees carrying partial trisomy 4q. In addition, the phenomenon of the partial trisomy 4q associate with 6p or Xp deletion have never been described before. Our finding further strengthens the supposition that mild phenotype in 4q duplication may be due to the spreading of X inactivation to the autosomal in derivation of chromosome X, which may provides valuable data for the prenatal genetic counseling.

## Data Availability

The datasets used and analysed during the current study available from the corresponding author on reasonable request.
